# A Brief Evaluation of a Project to Engage American Indian Young People as Agents of Change in Health Promotion Through Radio Programming, Arizona, 2009–2013

**DOI:** 10.5888/pcd13.150416

**Published:** 2016-02-11

**Authors:** Tara M. Chico-Jarillo, Athena Crozier, Nicolette I. Teufel-Shone, Theresa Hutchens, Miranda George

**Affiliations:** Author Affiliations: Athena Crozier, Theresa Hutchens, Miranda George, Hualapai Tribe, Peach Springs, Arizona; Nicolette I. Teufel-Shone, University of Arizona, Tucson, Arizona.

## Abstract

Young people can be valuable motivational resources for health promotion. A project implemented from 2009 through 2013 in a small American Indian community in northwest Arizona recruited American Indian young people aged 10 to 21 as agents of change for health promotion through radio programming. Thirty-seven participants were recruited and trained in broadcasting and creative writing techniques; they produced and aired 3 radio dramas. In post-project evaluation, participants were confident they could influence community behaviors but thought that training techniques were too similar to those used in school activities and thus reduced their drive to engage. Effective engagement of young people requires creativity to enhance recruitment, retention, and impact.

## Objective

Young people as agents of change can be a motivational resource for a community and promote positive development and resilience among young people (1). Young people are “influential in an adult’s social environment through multiple modes and across diverse cultural settings through communication and health behavior modeling” (2). Creating a place for and encouraging young people to actively participate in research and intervention processes builds skills and empowers them in their own well-being and the well-being of their community (1,3).

In many American Indian communities, lifestyle-related chronic disease is a leading cause of premature morbidity and mortality and requires innovative intervention to change risk factors supported by local norms, for example, acceptance of unhealthy food habits and regular sedentary behavior (4,5). To be effective, health promotion must be relevant to the sociocultural context and appealing to the target population. Radio programming is effective in influencing socially shared perceptions or social norms (6).

The objective of our project was to engage young people in an American Indian community to create locally relevant stories of a fictitious family working to make lifestyle changes and to share the stories with the community through a local radio station. 

## Methods

The project was conducted from October 2009 through December 2013 with participation of the Hualapai Tribe. The tribal reservation consists of approximately 1 million acres in rural northwest Arizona. Approximately 1,700 reservation residents (95% American Indian; 80% enrolled Hualapai tribal members) live in Peach Springs, Arizona, the primary residential and business community on the reservation. An estimated 40% of reservation residents are aged 18 years or younger.

Project steps involved recruitment and training of participants, story development and delivery, and assessment of impact on participants themselves and on community residents. In March 2010, participants were recruited from the local Boys & Girls Club and from the community-at-large through flyers and by word of mouth. Initially, any young person aged 12 to 21 years who had parental consent or could provide consent himself or herself (those aged >18 years) was eligible to participate. Because of initially poor recruitment outcomes, possibly resulting from attendance at off-reservation schools and competition with other activities, such as school sports, the minimum age was lowered to 10 years in June 2010. 

From July 2010 to December 2011, participants attended after-school and summer-training sessions that consisted of 1) didactic education on the role of healthy lifestyle behaviors and food choices and regular physical activity in sustaining health and 2) interactive activities in radio broadcasting and creative writing. Participants used Fooducate (Fooducate, Ltd), an application for smartphones that scans product barcodes to show nutrition information and support healthy food choices. Onsite mentorship from an outside broadcasting professional and modules on public speaking and creative writing were used to enhance skills in story development, broadcast delivery, and technology. By July 2012, participants had completed 3 stories and recorded them in English (the participants’ primary language) for broadcast on the local radio station. To enhance recruitment and retention, incentives such as after-school snacks and iTunes music cards, were offered to those who attended trainings sessions and participated in activities.

Evaluation consisted of 2 semistructured focus groups that were completed after the project ended in July 2013 and allowed participants to discuss their influence in the community and to reflect on their overall participation. Participants and their parents provided consent for participation in the focus groups, which were conducted in English and recorded through note taking. Notes were analyzed for content and themes using a multi-investigator consensus technique (5,7).

Project procedures and protocol were approved by the Hualapai Tribal Council and the Office of Human Subject Protection at the University of Arizona.

## Results

Sixty-five members of local Boys & Girls Club received information on the project. Throughout the 30 months of program implementation (training, story development, and broadcast education), 37 young people participated in the program; their ages ranged from 10 to 21 years.

In July 2012, each of the 3 dramas was aired twice per week during 3 consecutive weeks. The recorded broadcasts were enhanced with improved sound technology and re-aired in October 2012. The story followed a family consisting of young people, parents, grandparents, aunts, and uncles as they discussed opportunities for being physically active (eg, walking on popular foot paths, using the local fitness center) and shopped for food and ate fast food off the reservation. Characters were portrayed as change agents, early adopters, later adopters, and change resistors. After the stories were aired, the participants stayed engaged in radio program development and developed weekly radio programs that addressed health and nonhealth–related topics.

In July 2013, focus group participants (n = 11) indicated that they realized their ability to influence the community to make healthy lifestyle changes. They also believed that the local radio station was beneficial in informing the community about health issues because of the reach of the dramas and other radio programming. Half were confident they could motivate their community to be more active through their drama performance. 

Participants agreed they gained new skills in radio broadcasting. They enjoyed being able to create, record, and broadcast their own radio dramas. However, they felt the instructional components were similar to those used in school activities and thus negatively affected their interest in the training and the project as a whole. They indicated they would be more interested if more time were allotted to radio broadcasting and equipment use. Participants also indicated that the program could be improved by employing participants at the radio station in paid positions during the summer. 

The steps taken to enhance recruitment and retention midway through the project were moderately successful. One unanticipated positive outcome was that because of their enthusiasm for broadcasting, participants were inspired to develop other programs (interviews with community members, film reviews, and sports news programs) that were not directly related to the goals of the program.

## Discussion

For this project, young people were motivated to become active change agents in their community. One lesson learned was that nonschool–based training for young people needs to balance the goal of education and skill building with fun. In the initial stage of training on broadcast and story development, participants were required to spend significant amounts of time working around a table. This instructional method did not captivate their interest. Strategies to engage young people more effectively might include integrating interactive activities that would allow them to explore their creative interests and budgeting for an hourly wage so that participants could be paid.

The lessons learned in recruitment, retention, and training in this small American Indian community may be relevant to other settings. Engaging young people as change agents is heralded as an effective public health strategy (8), but doing so requires strategies that honor their competing requirements and interests (ie, school, sports, and other extracurricular activities) and integrates their motivation to improve their community in an entertaining way.
